# Supplementary material for: MetaRibo-Seq measures translation in microbiomes
Source: Nat Commun. 2020 Jun 29;11:3268. doi: 10.1038/s41467-020-17081-z (PMC7324362; doi:10.1038/s41467-020-17081-z)
Supplement: Supplementary file 10 — Supplementary Data 7 [file 41467_2020_17081_MOESM10_ESM.zip › File2/Confidence_High_Taxonomy/94618_out.krona.html]

Javascript must be enabled to view this page.

members
magnitude
magnitudeUnassigned
count
unassigned
taxon
rank

94618\_out

4

4
2759
superkingdom

kingdom
4751
4

4

SRS098571\_contig\_number\_64059
subkingdom
1
451864

4890
phylum
3

3
147537
subphylum

3
4891
class

4892
order
3

3
family
4893

3
71245
genus

species
655095
3

SRS051031\_contig\_number\_11313SRS053356\_contig\_number\_contig-100\_2400.136086SRS1041145\_contig\_number\_contig-100\_2801.181141
